# Supplementary figures and images for: Optimization and evaluation of a two-stage chromogenic assay procedure for measurement of emicizumab plasma levels
Source: PLoS One. 2022 Jul 14;17(7):e0271330. doi: 10.1371/journal.pone.0271330 (PMC9282532; doi:10.1371/journal.pone.0271330)

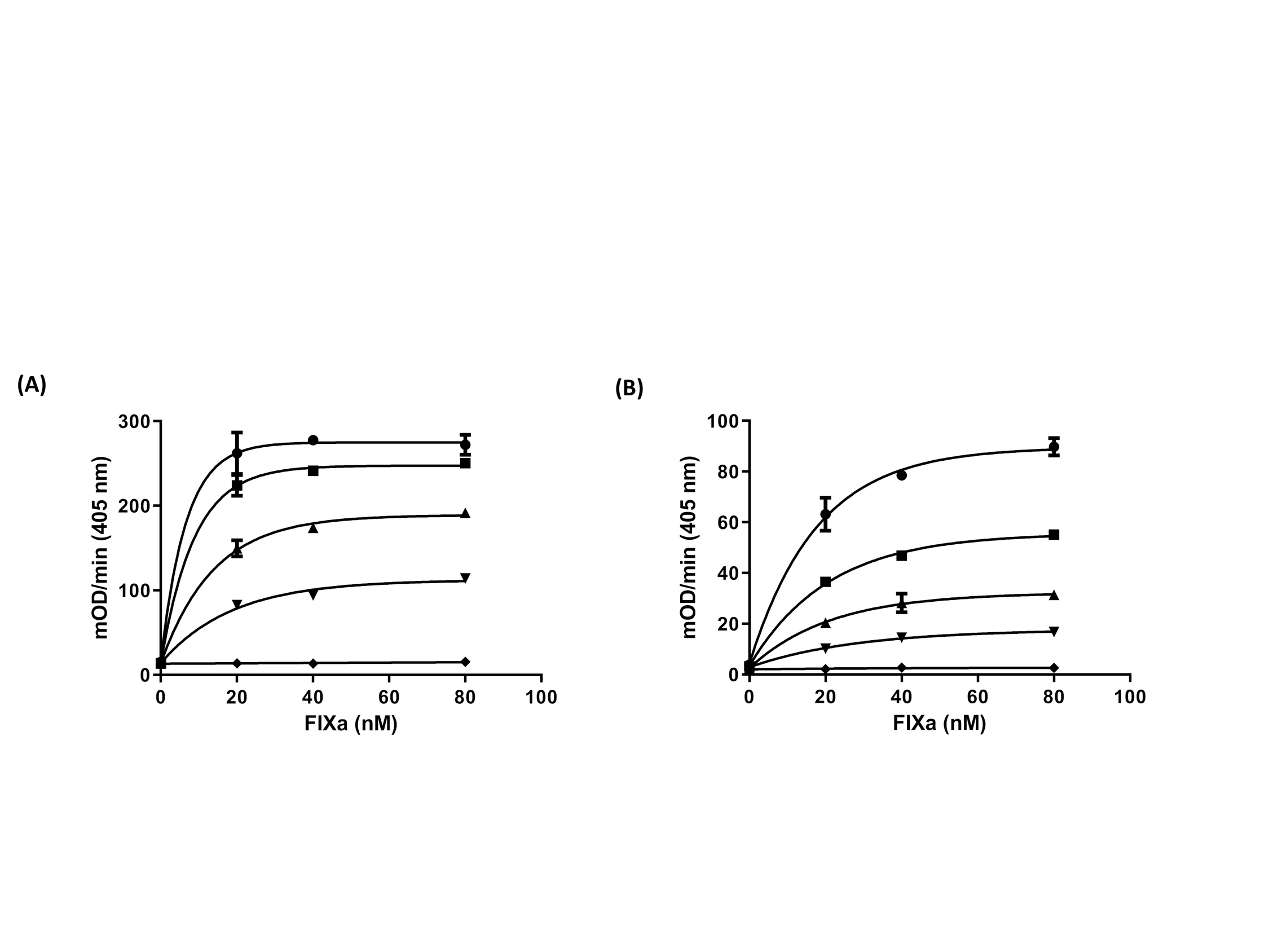

Supplement: S1 Fig — Twenty-five microliter of different concentrations of FIXa were mixed with 25 μl of various concentrations of FX in the presence of 100 μg/ml (A) or 10 μg/ml (B) emicizumab. FXa generation efficiency was measured after addition of 25 μl of the chromogenic substrate S-2765 (500 nM). Substrate conversion was measured at 405 nm using a microtiter plate reader. Data were collected in duplicate and showed as the means ± s.d. (TIF) [file pone.0271330.s001.tif]

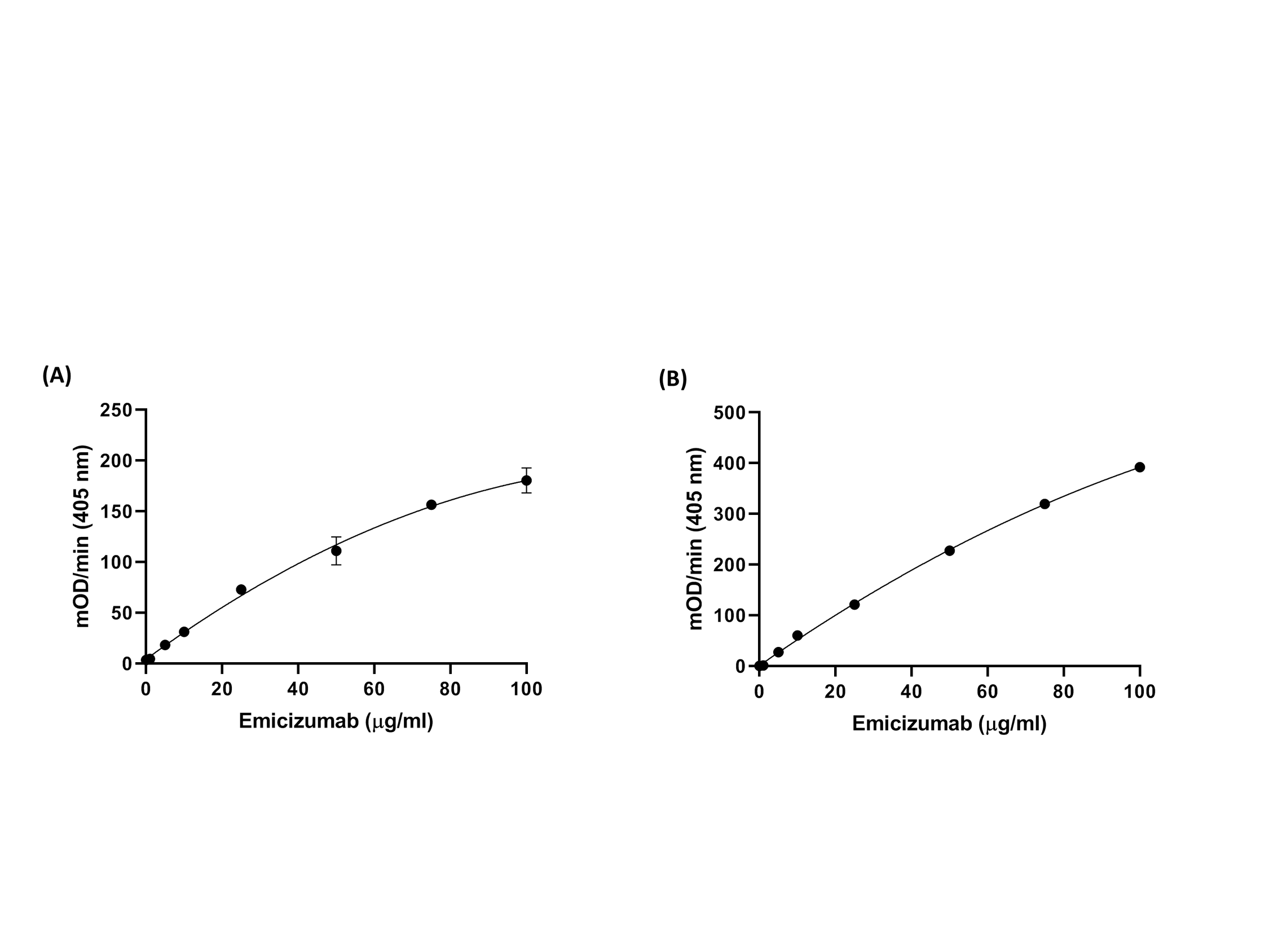

Supplement: S2 Fig — Typical standard curves resulted from different emicizumab concentrations in (A) manual and (B) automated format. Data were collected for manual configuration in triplicate and for automated configuration in duplicate showed as the means ± s.d. (TIF) [file pone.0271330.s002.tif]
