# Supplementary material for: Optimization and evaluation of a two-stage chromogenic assay procedure for measurement of emicizumab plasma levels
Source: PLoS One. 2022 Jul 14;17(7):e0271330. doi: 10.1371/journal.pone.0271330 (PMC9282532; doi:10.1371/journal.pone.0271330)
Supplement: Attachment — Submitted filename: Response to reviewers.pdf [file pone.0271330.s003.pdf]

Point-by-point response to reviewers

Submission ID: **PONE-D-22-10383**

Optimization and evaluation of a two-stage chromogenic assay procedure for measurement of emicizumab plasma levels

Dear Editor,

Here we would like to express our sincere thanks for considering our manuscript to be published in “PLOS ONE”. Please find enclosed a point-to-point response to the reviewers’ comments. We would like to emphasis that the additional journal requirements number 1 to 3 are addressed in the main text.
We hope that the revised version is suitable for publication.

Sincerely yours,
Dr. Nasim Shahidi Hamedani

RESPONSE TO REVIEWERS’ COMMENTS

General authors’ comments:
We would like to thank the reviewers for their critical evaluation of our manuscript. Applying mentioned changes helped us to improve the scientific impact of our manuscript. All the changes are made using tracked changes.

Comment #1, Reviewer #1:
The study has been designed well.

Response: We would like to thank the positive evaluation of the reviewer.

Comment #2, Reviewer #1:
Different parts of manuscript have been written appropriately.

Response: We would like to thank the positive evaluation of the reviewer

Comment #3, Reviewer #1:
Which criteria were used for patient selection? Please explain completely.

Response: All plasma samples were obtained from adult and adolescent hemophilia A (HA) patients developing inhibitory antibody against FVIII and receiving subcutaneous injection of emicizumab. The patients having the residual activity of FVIII upper than 2% were excluded from the study. The source of the patients/donors was clarified as well. All the changes are indicated in the Material and Methods, Plasma samples section.

Comment #4, Reviewer #1:
How many healthy blood donors were selected for this study?

Response: Three healthy blood donors were selected for this study and the plasma samples obtained from these donors were subjected to the spike and recovery experiment. The results were included in the Table 2.

Comment #1, Reviewer #2:
The advantage of heat-inactivation should be explained more precisely.

Response: The heat-inactivation process is described in more details in the material and methods section and its importance is explained precisely in discussion part.

Comment #2, Reviewer #2:
The authors should explain why the patients' samples are too

Response: The comment is not described in full sentence. We estimate it as if the patients' samples are too small. This is the only limitation of our study that at the time of the study the number of hemophilia patients eligible to receive emicizumab was less in our hemophilia center but according to the Bland-Altman method agreement analysis, the automated emi-tenase and the mOSC-assay which were performed on 17 plasma samples obtained from patients under emicizumab treatment showed high correlation of results whereat relative differences did not exceed 25%. Increasing the sample amount can either improve the agreement between two methods and support more our findings or leave the agreement values the same but not decrease it. Therefore, we are convinced that the number of patients and the design of the study is sufficient to conclude that the two-stage chromogenic assay is in accordance with one-stage clotting assay to quantify the emicizumab plasma levels.

Comment #3, Reviewer #2:
Please specify if the chromogenic FXa method was a kinetic or an endpoint method.

Response: A kinetic measurement of substrate conversion for 5 minutes is added to the materials and methods section (line 149).

Comment #4, Reviewer #2:
What are "general chemicals". This should be specified when describing the details of the assays. Accordingly, the first sentence can be eliminated.

Response: The changes are applied.
